# Effectiveness of zygomatic-implant fixed rehabilitation for the atrophic edentulous maxilla: protocol for a systematic review and network meta-analysis

**DOI:** 10.1186/s13643-024-02561-w

**Published:** 2024-05-31

**Authors:** Anisha Rodrigues, Samer Abi-Nader, Robert Durand, Pierre Rompré, Amal Idrissi Janati, Saadet Atsu, Martin Morris, Elham Emami

**Affiliations:** 1 Faculty of Dental Medicine and Oral Health Sciences, McGill University, Montreal, Canada; 2 Faculty of Dental Medicine, Université de Montréal, Montreal, Canada; 3 Université de Montréal, Montreal, Canada; 4 Materials Engineering, McGill University, Montreal, Canada; 5 Schulich Library, Mcgill University, Montreal, Canada

**Keywords:** Zygomatic implants, Oral rehabilitation, Network meta-analysis

## Abstract

**Background:**

Atrophic edentulous maxilla is a debilitating condition caused by the progressive and irreversible bone resorption following loss of teeth, that results in bone of inadequate volume and density. This makes conventional implant therapy extremely challenging without complex reconstructive procedures. Several techniques such as sinus augmentation, short implants, and tilted implants have been used for the rehabilitation of the atrophic maxilla. In recent years, zygomatic implants have emerged as a graftless rehabilitation technique. However, few studies compare zygomatic-implant fixed rehabilitation with other fixed rehabilitation techniques. The existing body of evidence on zygomatic implants is largely based on clinical and disease-oriented outcomes.

**Methods:**

A network meta-analysis (NMA) will be conducted in order to compare the effectiveness of zygomatic-implant fixed rehabilitation with the other rehabilitation techniques. Experimental and observational studies comparing different implant-assisted fixed rehabilitation in adults with atrophic maxilla will be included. The primary and secondary outcomes will be patient’s satisfaction and quality of life respectively. Additional outcomes include the implant’s survival/success, and biological and prosthetic complications. An electronic search will be performed through various databases for articles in English and French, without time limits. Risk of bias will be assessed using the Revised Cochrane Risk-of-Bias tool for randomized controlled trials, and ROBINS-I for non-randomized and observational studies. Two independent reviewers will screen the titles and abstracts and extract data. Any discrepancy between reviewers will be discussed and resolved through consensus or with the help of a third reviewer.

Pairwise meta-analyses will be performed using a random effects model. *I*^2^, *τ*^2^, transitivity, subgroup/meta-regression analyses will assess and explain heterogeneity and distribution of effect modifiers. A network plot will be created to connect the different interventions directly and indirectly. Interventions will be ranked using the surface under cumulative ranking curve. Confidence in the results of the NMA will be assessed using the Grading of Recommendations Assessment, Development and Evaluation (GRADE).

**Discussion:**

This study will be the first to assess the effectiveness of zygomatic-implant fixed rehabilitation for the atrophic maxilla using NMA. The evidence obtained will aid clinical decision-making and will advance the knowledge of the rehabilitation techniques for the atrophic maxilla.

**Systematic review registration:**

PROSPERO CRD42023353303.

**Supplementary Information:**

The online version contains supplementary material available at 10.1186/s13643-024-02561-w.

## Background

Edentulism (total tooth loss) is a serious life event and is considered to be the “final marker of disease burden for oral health” [1]. It is estimated that worldwide, 276 million people suffer from edentulism [2]. Among dental diseases, severe tooth loss has caused the highest reduction in global work productivity, amounting to $126.67 billion in 2015 [3].

One of the most important consequences of edentulism is a complex biophysical process of bone resorption that gradually progresses into an irreversible and debilitating condition of jaw atrophy [4]. In the maxilla, the bone resorption results in a short, narrow, or knife-edge residual alveolar ridge [5, 6] with low trabecular density cancellous bone and very thin or absent cortical bone [7]. In the posterior maxilla, the pneumatisation and enlargement of the maxillary sinus further reduces bone volume, which along with the bone resorption, results in a severely atrophic maxilla [8]. Conventional implant-supported fixed rehabilitation of the atrophic edentulous maxilla is challenging due to inadequate bone for implant anchorage [9].

In the recent past, advances in implant dentistry have provided clinicians with several options to address the complexities of rehabilitation of the atrophic maxilla [10–12]. Sinus augmentation with bone grafts is a commonly used technique for atrophic maxillary rehabilitation, with autogenous bone grafts being considered the gold standard [13]. A systematic review and meta-analysis of randomized controlled trials reported an implant survival rate of 98.7% with sinus lift and bone graft procedures in partially and completely edentulous atrophic maxilla [14]. However, this technique involves risks of complications and patient morbidity such as uncontrolled graft resorption and graft rejection, injury to the adjacent anatomic structures during graft harvesting, infection, delayed functional loading, and increased treatment costs [15]. To overcome these challenges, several graftless techniques have been proposed. These techniques are reported to be less invasive, involve less complex clinical procedures, reduce patient morbidity and treatment costs, and offer the possibility of immediate-loading, with faster rehabilitation [8, 12, 16].

Zygomatic implant rehabilitation is one of the graftless techniques that was introduced as a viable treatment option for the atrophic maxilla [16]. Tomographic studies have shown that the zygomatic bone density provides adequate anchorage of long implants to support a cross-arch fixed dental prosthesis [17]. Literature has reported high zygomatic implant survival rates that ranged from 95.2 to 100% [18]. When compared with sinus augmentation and delayed placement and conventional loading of dental implants, a randomized controlled study demonstrated shorter rehabilitation time (1.3 days versus 444.3 days) and fewer zygomatic implant and prosthesis failures at 1 year post-loading [19].

Previous systematic reviews and meta-analyses have assessed treatment outcomes with zygomatic implant rehabilitation [20–23]. However, the evidence does not allow optimal decision-making as it is largely based on clinical outcomes. The evidence from patient-reported outcomes is limited. Moreover, the evidence from these reviews is largely based on single-arm studies. Few studies have assessed the comparative effectiveness of zygomatic implant rehabilitation, due to which there is uncertainty as to whether zygomatic implant-fixed rehabilitation offers a distinct advantage over other techniques. The Network Meta-Analysis (NMA) offers the ability to overcome this limitation as it can estimate treatment effects of multiple treatments in a single analysis when head-to-head comparisons are scarce by combining direct evidence from clinical studies and indirect evidence from within the study network [24]. Furthermore, a hierarchy or “rank” for the techniques can be established [25]. The treatment rankings along with certainty estimates and level of evidence can better aid in the interpretation of the results that could help clinicians in their routine clinical practice and decision-making [26].

## Objectives

This systematic review aims to answer the following question: in patients with edentulous atrophic maxilla, how effective is zygomatic-implant fixed rehabilitation in comparison to other implant-supported fixed rehabilitation techniques with regard to patient-reported outcomes, implant survival, complications, and treatment costs?

## Methods

This protocol has been registered in PROSPERO (International Prospective Register of Systematic Reviews), under registration number CRD42023353303.

The protocol has been prepared in accordance with the Preferred Reporting Items for Systematic Review and Meta-Analysis Protocols, 2015 (PRISMA-P) [27] (Additional file 1).

The network meta-analysis will be conducted and reported according to the PRISMA-NMA extension statement [28].

### Eligibility criteria

The following criteria will be used to identify studies to be included in this review.

### Study design

Experimental studies (randomized controlled trials, non-randomized trials) and observational studies that compare outcomes of interest of at least two fixed rehabilitation techniques for atrophic maxilla will be included. Review articles, expert opinions, case reports, case series and publications using duplicated data will be excluded.

### Participants

Studies that enrolled completely edentulous adults (above 18 years of age) with an atrophic maxilla (< 8 mm bone in the posterior maxilla) will be included.

### Interventions

Zygomatic implants (combination of 2 zygomatic implants with regular implants in the anterior maxilla, or 4 zygomatic implants placed bilaterally).

### Comparators

1. Conventional or short implants with sinus elevation with or without bone grafts,
2. Tilted implants,
3. Any additional relevant techniques found during the literature search will be included in the review.

### Outcome(s)

The primary outcome will be patient satisfaction. We will use data measured with validated instruments such as the visual analog scale (VAS) or Likert scale.

The secondary outcome will be quality of life, which has been assessed using validated instruments.

Additional outcomes include implant survival and success, and biological and prosthetic complications.

### Time

Studies that have assessed outcomes with a minimum follow-up of 6 months after functional loading will be included in the review.

### Setting

Studies conducted in any dental care centers will be included.

### Language

Articles in English and French will be included in the review.

### Information sources

A comprehensive electronic search through MEDLINE, EMBASE, Web of Science, and Epistemonikos will be performed without limits on publication date. In addition, 3 clinical trial registries, Cochrane Central Register of Controlled Trials (CENTRAL), ClinicalTrials.gov, and WHO International Clinical Trials Registry Platform will be searched to identify completed and ongoing studies. Authors will be contacted to inquire about the status of their studies if the electronic search fails to identify them. Furthermore, hand-searching in relevant journals and reference sections of the included articles and previous systematic reviews on the topic will be conducted to identify any studies missed by the electronic search. To maximize the sensitivity of the search, no language restrictions will be applied to the search strategy.

### Search strategy

A draft version of the search strategy has been developed by members of the research team with expertise in systematic reviews and relevant clinical experience, and an expert librarian at McGill University, using Medical Subject Headings (MeSH), EMTREEs, and relevant text words. A draft of the MEDLINE search strategy can be found in Additional file 2. The finalized draft will be adapted to other databases using proper syntax, controlled vocabulary, and subject headings.

### Data selection

The identified articles from search results will be transferred to EndNote X9 software (Clarivate Analytics, PA, USA). Title and abstracts will be screened using Covidence (Veritas Health Innovation, Melbourne, Australia). Two independent reviewers will screen the titles and abstracts using the inclusion criteria. The process of data selection will be pilot-tested in 10% of randomly selected included studies. Reviewer’s agreement on study eligibility will be assessed using Cohen’s Kappa coefficient. Any discrepancy between reviewers will be discussed and resolved through consensus. The opinion of a third reviewer will be sought if consensus cannot be reached. Studies that do not fulfill the review criteria will be eliminated.

### Data collection process

Two reviewers will perform data extraction independently using a pre-established, electronic data extraction sheet in Microsoft Excel. The data extraction sheet will be pilot-tested on 5 randomly selected studies and amended if required. To ensure consistency, the data extraction process will be calibrated wherein two reviewers will independently extract data from 10% of the included studies. Any discrepancies in the extracted data will be discussed and a consensus will be reached before proceeding with the data extraction from the remaining studies.

### Data items

The following data will be extracted: title, authors, year of publication, journal, study design, patients’ characteristics (age, sex, comorbidities, smoking habits), number of participants, height of the residual ridge, interventions, comparisons, implant characteristics (length and diameter, surface treatment, morphological features), sinus elevation technique, type of bone grafts, opposing dentition, follow-up time, functional loading time, outcomes (patient satisfaction, quality of life, implant survival rate, complications), and study authors’ main conclusions. Study authors will be contacted to obtain any missing data. Disagreements between reviewers will be resolved through discussions and any unresolved issues will be adjudicated by a third reviewer.

### Outcomes and prioritization

The primary outcome will be patient satisfaction.

The secondary outcome will be quality of life.

This review will focus on patient-reported outcomes as evidence from these outcomes is important in assessing the benefits and harm of treatment, assisting with treatment selection, and facilitating communication [29]. Evidence from patient-reported outcomes is crucial to develop strong recommendations (Level A, Strength of Evidence Taxonomy (SORT)) [30] and allow optimal clinical decision-making.

The additional outcomes will be clinical outcomes: implant survival rate, biological and prosthetic complications, and treatment costs.

### Risk of bias assessment

Two reviewers will independently assess each study. Disagreements between the reviewers over the risk of bias in particular studies will be resolved by discussion, with the involvement of a third reviewer, whenever necessary. The Revised Cochrane Risk-of-Bias tool for Randomized trials, Version 2.0 (RoB 2) [31] will be used to assess the risk of bias for randomized controlled trials under the five domains: (1) bias arising from the randomization process; (2) bias due to deviations from intended interventions; (3) bias due to missing outcome data; (4) bias in the measurement of the outcome; (5) bias in the selection of the reported result. The risk of bias will be classified under each domain as (1) low risk of bias (low risk of bias for all domains); (2) some concerns (some concerns for at least one domain but not at high risk of bias for any domain); (3) high risk of bias (high risk of bias in at least one domain or have some concerns for multiple domains that may substantially lower the confidence in the result).

For non-randomized and observational studies, the Risk of Bias In Non-randomized Studies–of Interventions (ROBINS-I) will be used [32] to assess the methodological quality under the following 7 domains: confounding, selection bias, bias in measurement classification of interventions, bias due to departures from the intended interventions, bias due to missing data, bias in measurement of outcomes and bias in selection of the reported result. The studies will be rated as low risk (low risk of bias for all domains), moderate risk (low or moderate risk of bias for all domains), serious risk (serious risk of bias in at least one domain, but not at critical risk of bias in any domain), and critical risk of bias (critical risk of bias in at least one domain). If there is insufficient information, the risk of bias will be classified as “no information”.

### Data synthesis

The descriptive synthesis will follow the guidance from the Centre for Reviews and Dissemination [33]. Text and tables will be used to summarize the characteristics of the included studies. Data synthesis will be performed on the assumption of transitivity, i.e., all studies are similar, except for the type of intervention, and that the participants are jointly randomizable (eligible to receive any treatment in the network). In addition, the effect modifiers (factors that induce heterogeneity and influence the treatment outcomes, e.g., age and gender) have to be evenly distributed across the network. Clinical advice will be sought to assess the plausibility of this assumption. Transitivity will be evaluated by assessing the distribution of the effect modifiers between the treatment groups using boxplots or percentages. The possible violation of transitivity due to factors such as functional loading time, and types of bone grafts will be evaluated through subgroup and meta-regression analyses.

The unit of analysis will be the patient, for patient-reported outcomes and clinical outcomes, and each implant for implant survival.

We will calculate the pooled odds ratios for dichotomous outcomes and pooled standard mean difference for continuous outcomes with a 95% confidence interval.

The analysis will consider three follow-up points: an early period (up to 2 years after immediate loading), a medium period (2–5 years), and a late period(> 5 years). All analyses will be performed using the R package (R Project for Statistical Computing, The R Foundation, Vienna, Austria).

### Pairwise meta-analysis

Standard meta-analysis for each pairwise comparison of the interventions will be performed using the random effects model. As this review will classify studies by surgical techniques, substantial heterogeneity is anticipated. Methodological heterogeneity will be explored by assessing the study quality. The clinical heterogeneity of the studies will be assessed by checking the baseline characteristics of patients, surgical approaches, types of bone grafts, implant characteristics, and time to function (immediate vs delayed loading). Statistical heterogeneity will be evaluated using visual inspection of the forest plots and quantified using the *I*^*2*^ and the tau square (*τ*^*2*^*)* statistic. *I*^2^ value < 40% suggests a non-significant amount of heterogeneity, 30% to 60% suggest moderate heterogeneity, 50% to 90% suggest substantial heterogeneity, and 75% to 100% represent considerable heterogeneity [34]. A *τ* ^2^ > 1 suggests substantial heterogeneity. The heterogeneity of the studies > 75% will be explored in subgroup analysis and meta-regression analysis.

### Network meta-analysis

NMAs with random effects will be performed using the *netmeta package* in R, version *4.1.3*. A network plot will be created to connect the different interventions directly and indirectly. The network plot consists of nodes, the size of which will be proportional to the number of participants for each intervention, and edges with thickness proportional to the number of studies comparing two interventions. The interventions will be clubbed under the following groups: zygomatic implants (combination of two zygomatic implants with regular implants in the anterior maxilla or four zygomatic implants); conventional implants with or without sinus elevation and bone augmentation; short dental implants with or without bone augmentation; and tilted implants. Studies that are not connected will be excluded from the network.

Consistency of the NMA, which refers to the statistical agreement between direct and indirect comparisons will be assessed. We will assess inconsistency for the network through the local and global approaches using the *netsplit* and *decomp.design* functions respectively.

Separate NMA of randomized and non-randomized studies will be performed to assess the agreement between them. Interventions will be ranked using the surface under the cumulative ranking curve (SUCRA) for the outcomes.

### Subgroup analysis

Subgroup analysis will be used to explore heterogeneity and/or inconsistency. If heterogeneity/inconsistency is identified, their possible sources based on the effect modifiers will be explored. If sufficient data is present, we will investigate the treatment effect based on the following factors: patient characteristics (e.g., gender, age), surgical approach, type of bone grafts, time to function (immediate or delayed loading), and study quality.

### Level of evidence

The Grading of Recommendations Assessment, Development, and Evaluation (GRADE) approach will be used to rate the certainty of evidence from the NMA [35, 36] based on the ratings of direct and indirect evidence. Based on the recent recommendations by the GRADE working group [36], the direct evidence will be assessed through the following criteria: risk of bias, inconsistency, indirectness, and publication bias. The certainty of direct estimates will inform the indirect estimates along with intransitivity. The certainty of network estimates will be down-rated in the presence of considerable imprecision or incoherence. The level of evidence will be classified under four levels: high level, moderate level, low level, and very low. The GRADE assessment will be conducted independently by two reviewers. Any disagreement will be discussed and resolved by consensus, or with the help of a third reviewer if necessary.

### Publication bias

We will use the “comparison-adjusted funnel plot” to identify possible small-study effects in the NMA [37].

### Differences between the protocol and the review

Any deviations from the protocol due to unanticipated issues will be reported in the final review.

## Discussion

The atrophic edentulous maxilla bears both clinical and economic implications to the patient and the clinician. As a result, newer, innovative techniques have been developed that are aimed at minimizing complications, ensuring patient comfort with a minimal time to function, and enhancing overall quality of life.

To date, there is a lack of definitive evidence regarding the most effective implant rehabilitation technique for the atrophic maxilla [16]. It is therefore crucial to comprehensively examine all existing evidence to guide clinicians and inform patients to determine the optimal technique for rehabilitation.

Previous reviews have indicated that zygomatic implant rehabilitation could meet the treatment requirements of patients [11, 16, 18]. Nonetheless, we anticipate that this network meta-analysis (NMA) will enhance the existing evidence by directly and indirectly comparing this technique with other treatment modalities.

This review will evaluate patient satisfaction as the primary outcome as it is considered a valid measure and an important predictor of treatment success and quality of care [38]. In contrast to clinical outcomes such as implant survival, morbidity, and complications, patient satisfaction reflects the patients’ perception of the treatment relative to their expectations and is dependent on the dentist-patient relationship, treatment affordability, and psychological and clinical factors [39, 40].

Though it is recommended that NMAs include RCTs for the best evidence, our review will include observational studies that can better reflect patient experiences and provide real-world evidence [41, 42]. Moreover, additional data can be obtained on competing therapies particularly when RCTs may not be feasible due to the complexity of the procedures [43]. Evidence from these studies can complement the evidence from RCTs and improve the precision of findings [44]. Another advantage is that adverse events, complications, and unintended effects of treatment can be better determined through these study designs [45]. However, the inclusion of observational studies in this review presents a potential limitation, as considerable heterogeneity between the primary studies might be encountered. Furthermore, these studies are susceptible to reduced internal validity due to the increased risk bias and confounding [46]. To address these limitations, both qualitative and quantitative assessments of heterogeneity will be performed with subgroup and meta-regression where required. The risk of bias will be carefully assessed and studies that account for confounding will be included in this review.

### Implications of the review

To our knowledge, no previous systematic review with NMA has been conducted on rehabilitation techniques for the atrophic edentulous maxilla. Given the burden of the condition, particularly in the elderly, this NMA will provide an important evidence base for clinicians to inform treatment decisions and direct future research in this field.

### Supplementary Information

Additional file 1. Preferred Reporting Items for Systematic Review and Meta-Analysis Protocols, 2015 (PRISMA-P) [27].

Additional file 2. Search strategy.

## Data Availability

Not applicable.

## Abbreviations

NMA: Network meta-analysis
PRISMA-P: Preferred Reporting Items for Systematic Review and Meta-Analysis Protocols 2015 statement
RCT: Randomised controlled trials
ROBINS-I: Risk Of Bias In Non-Randomized Studies-of Interventions
SORT: Strength of Evidence Taxonomy
SUCRA: Surface under the cumulative ranking curve
