# Supplementary material for: Effectiveness of zygomatic-implant fixed rehabilitation for the atrophic edentulous maxilla: protocol for a systematic review and network meta-analysis
Source: Syst Rev. 2024 May 31;13:146. doi: 10.1186/s13643-024-02561-w (PMC11140941; doi:10.1186/s13643-024-02561-w)
Supplement: Supplementary file 2 — Additional file 2. Search strategy. [file 13643_2024_2561_MOESM2_ESM.docx]

**Database:**
Ovid MEDLINE(R) (1996 to February 28, 2023)

| **#** | **Query** | **Results from 1 Mar 2023** |
| --- | --- | --- |
| 1 | Mouth, Edentulous/ or Jaw, Edentulous/ | 5,787 |
| 2 | (edentulous* or edentate or edentulism).tw,kf. | 11,098 |
| 3 | (complete adj3 tooth adj3 loss).tw,kf. | 73 |
| 4 | or/1-3 | 12,647 |
| 5 | exp Dental Implants/ | 25,971 |
| 6 | exp Dental Implantation/ | 16,708 |
| 7 | Dental Prosthesis/ | 1,426 |
| 8 | Dental Prosthesis, Implant-Supported/ | 9,643 |
| 9 | Dentures/ or Denture, Complete/ or Denture, Overlay/ | 6,337 |
| 10 | ((implant$ or restor* or rehabilitat*) and (dental* or oral*)).tw,kf. | 65,207 |
| 11 | (denture* or overdenture* or prosthes?s).tw,kf. | 77,511 |
| 12 | or/5-11 | 146,625 |
| 13 | 4 and 12 | 9,860 |
| 14 | exp Bone Resorption/ | 28,914 |
| 15 | ((bone or jaw or alveol* or sinus* or ridge*) adj5 (loss or resorp* or reabsorb* or atroph* or reduc*)).mp. | 88,087 |
| 16 | 14 or 15 | 92,335 |
| 17 | exp Maxilla/ or maxill*.tw,kf,hw. | 90,543 |
| 18 | 13 and 16 and 17 | 1,276 |
| 19 | "***CADTH CLINICAL TRIALS FILTER***".sm. | 0 |
| 20 | (Randomized Controlled Trial or Controlled Clinical Trial or Pragmatic Clinical Trial or Clinical Study or Adaptive Clinical Trial or Equivalence Trial or (Clinical Trial or Clinical Trial, Phase I or Clinical Trial, Phase II or Clinical Trial, Phase III or Clinical Trial, Phase IV or Clinical Trial Protocol) or Multicenter Study).pt. | 967,584 |
| 21 | Clinical Studies as Topic/ or exp Clinical Trial/ or exp Clinical Trials as Topic/ or Clinical Trial Protocol/ or Clinical Trial Protocols as Topic/ or Multicenter Study/ or Multicenter Studies as Topic/ or "Multicenter Study (topic)"/ or Randomization/ or Random Allocation/ or Double-Blind Method/ or Double Blind Procedure/ or Double- Blind Studies/ or Single-Blind Method/ or Single Blind Procedure/ or Single-Blind Studies/ or Placebos/ or Placebo/ or Control Groups/ or Control Group/ or Cross- Over Studies/ or Crossover Procedure/ | 1,332,895 |
| 22 | (random* or sham or placebo* or ((singl* or doubl*) adj (blind* or dumm* or mask*)) or ((tripl* or trebl*) adj (blind* or dumm* or mask*)) or (control* adj3 (study or studies or trial* or group*)) or (clinical adj3 (study or studies or trial*)) or (Nonrandom* or non random* or non-random* or quasi-random* or quasirandom*) or (phase adj3 (study or studies or trial*)) or ((crossover or cross-over) adj3 (study or studies or trial*)) or ((multicent* or multi-cent*) adj3 (study or studies or trial*))).ti,ab,hw,kf. or allocated.ti,ab,hw. or ((open label or open-label) adj5 (study or studies or trial*)).ti,ab,hw,kf. or ((equivalence or superiority or non-inferiority or noninferiority) adj3 (study or studies or trial*)).ti,ab,hw,kf. or (pragmatic study or pragmatic studies).ti,ab,hw,kf. or ((pragmatic or practical) adj3 trial*).ti,ab,hw,kf. or ((quasiexperimental or quasi-experimental) adj3 (study or studies or trial*)).ti,ab,hw,kf. or trial.ti,kf. | 3,208,622 |
| 23 | 20 or 21 or 22 | 3,208,959 |
| 24 | (random* or sham or placebo* or ((singl* or doubl*) adj (blind* or dumm* or mask*)) or ((tripl* or trebl*) adj (blind* or dumm* or mask*)) or (control* adj3 (study or studies or trial* or group*)) or (clinical adj3 (study or studies or trial*)) or (Nonrandom* or non random* or non-random* or quasi-random* or quasirandom*) or (phase adj3 (study or studies or trial*)) or ((crossover or cross-over) adj3 (study or studies or trial*)) or ((multicent* or multi-cent*) adj3 (study or studies or trial*))).ti,ab,hw,kf. or allocated.ti,ab,hw. or ((open label or open-label) adj5 (study or studies or trial*)).ti,ab,hw,kf. or ((equivalence or superiority or non-inferiority or noninferiority) adj3 (study or studies or trial*)).ti,ab,hw,kf. or (pragmatic study or pragmatic studies).ti,ab,hw,kf. or ((pragmatic or practical) adj3 trial*).ti,ab,hw,kf. or ((quasiexperimental or quasi-experimental) adj3 (study or studies or trial*)).ti,ab,hw,kf. or trial.ti,kf. | 3,208,622 |
| 25 | exp humans/ or exp human experiment/ | 14,057,229 |
| 26 | 24 not 25 | 763,736 |
| 27 | 23 not 26 | 2,445,223 |
| 28 | 18 and 27 | 443 |
| 29 | "***CADTH OBSERVATIONAL STUDIES FILTER***".sm. | 0 |
| 30 | Epidemiologic Methods/ or exp Epidemiologic Studies/ or Observational Studies as Topic/ or Clinical Studies as Topic/ or single-case studies as topic/ or organizational case studies/ | 2,758,771 |
| 31 | (Observational Study or Validation Studies or Clinical Study).pt. or (observational adj3 (study or studies or design or analysis or analyses)).ti,ab,kf. or cohort*.ti,ab,kf. or (prospective adj7 (study or studies or design or analysis or analyses)).ti,ab,kf. or ((follow up or followup) adj7 (study or studies or design or analysis or analyses)).ti,ab,kf. or ((longitudinal or longterm or (long adj term)) adj7 (study or studies or design or analysis or analyses or data)).ti,ab,kf. or (retrospective adj7 (study or studies or design or analysis or analyses or data or review)).ti,ab,kf. or ((case adj control) or (case adj comparison) or (case adj controlled)).ti,ab,kf. or (case-referent adj3 (study or studies or design or analysis or analyses)).ti,ab,kf. or (population adj3 (study or studies or analysis or analyses)).ti,ab,kf. or (descriptive adj3 (study or studies or design or analysis or analyses)).ti,ab,kf. or ((multidimensional or (multi adj dimensional)) adj3 (study or studies or design or analysis or analyses)).ti,ab,kf. or (cross adj sectional adj7 (study or studies or design or research or analysis or analyses or survey or findings)).ti,ab,kf. or ((natural adj experiment) or (natural adj experiments)).ti,ab,kf. or (quasi adj (experiment or experiments or experimental)).ti,ab,kf. or ((non experiment or nonexperiment or non experimental or nonexperimental) adj3 (study or studies or design or analysis or analyses)).ti,ab,kf. or (prevalence adj3 (study or studies or analysis or analyses)).ti,ab,kf. or case series.ti,ab,kf. or case reports.pt. or (case adj3 (report or reports or study or studies or histories)).ti,ab,kf. | 4,248,087 |
| 32 | 30 or 31 | 5,354,604 |
| 33 | 18 and 32 | 918 |
| 34 | 28 or 33 | 1,028 |
